# Monitoring and Control of *Aedes albopictus*, a Vector of Zika Virus, Near Residences of Imported Zika Virus Patients during 2016 in South Korea

**DOI:** 10.4269/ajtmh.17-0587

**Published:** 2017-11-13

**Authors:** Kyu-Sik Chang, Gi-Hun Kim, Young-Ran Ha, Eun Kyeong Jeong, Heung-Chul Kim, Terry A. Klein, Seung Hwan Shin, Eun Jeung Kim, Seung Jegal, Se Jin Chung, Young-Ran Ju, Young Mee Jee

**Affiliations:** 1 Korea Center for Disease Control and Prevention, Osong, Chungbuk, Republic of Korea;; 2 5th Medical Detachment, 168th Multifunctional Medical Battalion, 65th Medical Brigade, Unit 15247, USAG Yongsan, Seoul, Korea;; 3 Medical Department Activity-Korea/65th Medical Brigade, Unit 15281, USAG Yongsan, Seoul, Korea;; 4 Seoul Metropolitan Government Research Institute of Public Health and Environment, Seochogu, Seoul, Republic of Korea;; 5 Gangwon Institute of Health and Environment, Shinbuk, Gangwon, Republic of Korea;; 6 Incheon Institute of Health and Environment, Joonggu, Incheon, Republic of Korea

## Abstract

Zika virus (ZIKV) is an arthropod-borne virus mainly transmitted by *Aedes* species. A total of nine of the 16 imported ZIKV reported cases during the mosquito season in the Republic of Korea (ROK), following the return of local nationals from foreign ZIKV endemic countries, were surveyed for *Aedes albopictus*. Surveillance and vector control of *Ae. albopictus*, a potential vector of ZIKV, and related species are critical for reducing the potential for autochthonous transmission in the ROK. Surveillance and vector control were coordinated by Korean Centers for Disease Control & Prevention (KCDC) and conducted by local health authorities within 200 m of imported ZIKV patients’ residences. After diagnosis, thermal fogging (3 × week × 3 weeks), residual spray for homes and nearby structures (1 × week × 3 weeks), and larval control (3 × week × 3 weeks) were conducted in accordance with national guidelines developed by KCDC in early 2016. Of the nine residences surveyed using BG Sentinel traps, *Ae. albopictus* trap indices (TIs) for the three (3) patients’ residences located near/in forested areas were significantly higher than the six patients’ residences located inside villages/urban areas or low-lying farmland without trees. Overall, *Ae. albopictus* TIs in forested areas decreased by 90.4% after adult and larval control, whereas TIs decreased by 75.8% for residences in nonforested areas. A total of 3,216 *Aedes* and *Ochlerotatus* spp. were assayed by real-time polymerase chain reaction for ZIKV, dengue, and chikungunya virus. Both species collected before and after vector control were negative for all viruses. Vector control within 200 m of residences of imported ZIKV patients, conducted in accordance with established guidelines, may have effectively reduced human–mosquito–human transmission cycle by competent vectors in South Korea.

## INTRODUCTION

Flaviviruses, for example, dengue virus (DENV) and Zika virus (ZIKV), and Alphaviruses, for example, chikungunya virus (CHIKV), attracted little attention until 2000 when increasingly higher numbers of infections were reported in urban settings throughout much of the tropical and subtropical areas of the world. While *Aedes aegypti* is the primary vector in most areas, *Aedes albopictus*, which is present in relatively high numbers in forested areas of the Republic of Korea (ROK), as well as limited urban environments (e.g., where used tires are improperly stored), is considered a secondary vector. *Aedes albopictus* gained considerable attention in 2016 when > 100 imported cases of DENV were reported annually since 2010 in the ROK as travelers returned home from endemic countries,^1^ in addition to 16 imported cases of ZIKV in 2016.

ZIKV, a member of the Family Flaviviridae, was first isolated in Uganda, Africa, from monkeys in 1947 and from *Aedes africanus* in 1948.^2^ While there were occasional cases and outbreaks, its recent adaptation to efficiently infect *Ae. aegypti*, *Ae. albopictus*, and several other *Aedes* spp. in the Pacific Islands has led to its widespread dispersal and outbreaks in many of the tropical and semitropical regions of the world, including nine countries in Asia, 47 in Central and South America, one in North America, 10 in Oceania, and two in Africa, with the potential to spread to temperate regions during “mosquito seasons”.^3–6^ ZIKV is a monkey-mosquito-monkey forest cycle zoonotic disease that has been adapted to an urban *Ae. aegypti*-man-*Ae. aegypti* (and related *Aedes* species) cycle with the potential for emergence in nonendemic areas where competent mosquito vectors (e.g., *Ae. albopictus*) are present.^7^ Worldwide, *Ae. aegypti*, and *Ae. albopictus* to a lesser degree because of its broader range of hosts, have been identified as primary and secondary vectors of ZIKV.^8^ Vector competence studies of *Ae. albopictus* and *Ae. aegypti* exposed to the same mice infected by ZIKV showed that the viremia levels in mice produced disseminated infections. However, *Ae. albopictus* infection rates were more dose dependent, requiring higher blood meal titers than titers required for *Ae. aegypti* infections.^9^

Unfortunately, as with other arboviruses, for example, West Nile virus and CHIKV, there are no government approved human vaccines or other specific treatments for ZIKV.^10^ Therefore, it is essential to implement effective vector control for target species near residences where imported ZIKV-infected patients in the ROK are identified to decrease the potential human-vector-human transmission risks during the late spring to fall mosquito season. An example of this type of approach occurred in Chiba City, Japan, in 2014, when emergency vector surveillance and control strategies of *Ae. albopictus* were implemented due to local transmission of DENV. The result was that *Ae. albopictus* populations were significantly reduced after adult control strategies were implemented, which also reduced the risks for future transmission of DENV.^11^

A total of 16 imported ZIKV cases, including one asymptomatic case, were identified from January to October 2016 in the ROK. Nine of the cases were reported during the primary mosquito season from June to October.^12^ All ZIKV cases were attributed to transmission by mosquitoes during their travels to foreign ZIKV endemic countries, with 77% of the cases attributed to travel to Southeast Asia (Philippines, Vietnam, and Thailand).^13^ While the Korean Centers for Disease Control & Prevention (KCDC) has established vector control guidelines for vivax malaria and Japanese encephalitis that are endemic in the ROK, there were no established guidelines for vector control of nonendemic vector-borne diseases, for example, ZIKV, DENV, and CHIKV, imported from tropical and subtropical countries where they are endemic. To reduce the potential for autochthonous transmission of imported mosquito-borne viruses during the mosquito season in the ROK, the KCDC established guidelines in 2016 for the control of *Ae. albopictus*, the primary vector in Korea.^4^ However, the efficacies of these guidelines have not been fully evaluated.

## METHODS

### Patient management.

Returning local nationals and foreigners from endemic countries who demonstrated high fever, rash, and myalgia or arthralgia entering the country or reporting symptoms shortly after arrival were tested for ZIKV. All confirmed ZIKV patients were hospitalized for a period of a few hours to 1 day. The origin of infection was determined through an epidemiological investigation conducted during patient hospitalization. Patients were advised not to enter or go near forested areas, for example, hiking, and to use bed nets while resting/sleeping where mosquitoes were present. Patients who worked or resided near/in forested areas were informed that they should wear long pants and long-sleeved shirts and use repellents for a minimum of 3 weeks after laboratory confirmation of ZIKV infection. Residences were surveyed, and torn window screens repaired and treated daily for 3 weeks with 0.5% permethrin applied by a 2-gallon sprayer.

### Mosquito surveillance near ZIKV patients’ residences.

The KCDC requested the cooperation of the ZIKV-infected patients and their family members in the establishment of mosquito surveillance and control activities that were conducted by the Local Public Health Centers (LPHCs) and Local Environment and Health Institutes (LEHIs). The KCDC provided training to members of the LPHIs and LPHCs on surveillance and control procedures to ensure a harmonized approach by the various groups. After confirmation of ZIKV patient infections during the mosquito season from June to October, the LPHCs conducted surveillance for a 24-hour period twice weekly for 3 weeks when it was not raining at six sites within 200 m of each of the ZIKV patient residences that targeted *Ae. albopictus* using BG Sentinel^®^ traps (Biogents, Regensburg, Germany) (Table 1). After each collection, LPHC personnel placed the adult mosquitoes in a Styrofoam cooler with dry ice, and then transported them to the LEHI laboratories where they were maintained at −70°C. Subsequently, the mosquitoes were transferred to a chill-table (−10°C) and identified to species. *Aedes* and *Ochlerotatus* species were placed in 2-mL cryovials, by species and date and location of the collection, and later assayed for the detection of ZIKV, DENV, and CHIKV.

**Table 1:** Larval and adult control of *Aedes albopictus* within 200 m near imported Zika virus patients’ residences during mosquito season in the Republic of Korea, 2016

Case number	Date of collection*		Temperature (°C)†		Humidity (%)†	
	Pre‡	Post	Pre	Post	Pre	Post
6	June 30	July 21	24.6	28.3	78.1	61.3
7	July 9	July 30	26.3	30.1	65.4	62.3
8 (R) and 8 (O)§	July 13	August 3	26.3	29.1	69.1	65.4
9	July 28	August 18	29.7	28.9	79.0	79.3
10	August 19	September 9	28.9	27.6	69.5	71.7
11	August 26	September 16	24.6	23.8	82.9	84.8
12	September 14	October 5	24.5	23.3	64.1	82.0
13	September 19	October 10	22.0	21.5	69.3	55.1
14	September 23	October 14	21.4	20.6	57.9	61.8

* Date = date of mosquito collection pre and post-control.

† Temperature and Humidity = daily mean temperature and humidity.

‡ Pre or Post = before and after mosquito control.

§ Cases 8 (R) and 8 (O) are the same patient. Case 8 (R) represents case 8 residence, and case 8 (O) represents case 8 office. Vector control was conducted only near the patient’s residence. Patient 8 (O) reported mosquito bites at his workplace, but only mosquito surveillance was conducted.

### RNA extraction of mosquito pools for virus detection.

Mosquito pools were homogenized in 750 μL of RPMI 1640 medium with Tissue Lyser II (QIAGEN, Valencia, CA). The homogenate was centrifuged at 13,000 rpm for 1 minute at 4°C and then 140 μL of the supernatant was subjected to viral RNA extraction. The remaining homogenate was maintained at −70°C for future use. Total RNA was extracted from the mosquito homogenates using Trizol reagent (Invitrogen, Grand Island, NY), in accordance with the manufacturer’s instructions, then resuspended in 50 μL of RNase-free water containing 10 units of RNasin^®^ Plus RNase Inhibitor (Promega, Madison, WI), and then stored at −70°C until used.

### Detection of ZIKV, DENV, and CHIKV in *Aedes* and *Ochlerotatus* spp. mosquitoes.

A one-step real-time reverse transcription-polymerase chain reaction (RT-PCR) assay for the detection of ZIKV, DENV, and CHIKV in mosquitoes was conducted using a QIAamp Viral RNA Mini kit (QIAGEN Inc., Germantown, MD) for RNA extraction and PowerChek^™^ Real-time PCR kit (KogenBiotech Co., Ltd, Seoul, Korea) for ZIKV/DENV/CHIKV in accordance with manufacture instructions. Species-specific multiplex primers (forward and reverse) were used to detect ZIKV (JOE), DENV (FAM), and CHIKV (ROX) in accordance with manufactures directions. RT-PCR was performed with 4.2-μL primer/probe mix, 10-μL reaction buffer, 0.8-μL, enzyme mix, and 5-μL template RNA. RT-PCR conditions for each reaction were 30 minutes at 50°C, followed by 10 minutes at 95°C, then 15 seconds at 95°C for 40 cycles, and final annealing temperature for 1 minute at 60°C. For positive controls, 5 μL of control mixture (Tris EDTA buffer and Partial fragments of ZIKV, DENV, and CHIKV) was added instead of sample RNA, and for negative control amplification, 5 μL of nuclease distilled water was added instead of sample RNA.

### Adult and larval control of *Aedes* and *Ochlerotatus* spp. near ZIKV patients’ residences.

Nine government ministers held a ZIKV countermeasure meeting in February 2016, where they checked preparatory attitudes and assigned roles to each minster for ZIKV vector control. Guidelines were developed by the KCDC for monitoring and implementation of control measures for *Ae. albopictus* (first edition) and distributed to local governments and related ministers. Vector control agencies of local governments were educated by vector control experts in accordance with the recently developed KCDC guidelines. However, guidelines were drafted based only on peer-reviewed published articles and documented references, where, in many cases, the control efficacy in the field had not been adequately evaluated.

*Aedes albopictus* vector control guidelines, established by the KCDC based on BG Sentinel trap indices (TIs) (number of specimens collected/trap night), were applied for *Aedes* and *Ochlerotatus* species control after the confirmation of ZIKV infections among residents returning from endemic areas during the mosquito season (ZIKV cases 6–14, excluding case 8–1) (Table 2). Pesticides were applied using a portable thermal fogger, SS-fog^®^ 150 or 180 (Seshin industrial Co., Seoul, ROK) three times weekly for a period of 3 weeks from 18:00 to 20:00 within 200 m near ZIKV patients’ residences, which included vegetable gardens, ivy-covered walls, and forested areas in parks. Insecticides applied using a thermal fogger included: pyrethroids, for example, Vegadelta^®^ 25EC (2.5% deltamethrin) (Best tech Korea, Suwon, ROK), Greenbug plus^®^ EC (5% etofenprox) (Sip ja sung Inc., Youngin, ROK), and Triplebifen^®^ (5% bifenthrin) (Glory Technical, Yeonggwang, ROK) in accordance with label directions. Pyrethroids were also applied to the walls of ZIKV patients’ homes and nearby structures within 200 m using a 2.6 gallon portable air-compressed residual sprayer (Clover air compressed sprayer^®^ TH-1200; Taehwan Co., Gwangmyeong, ROK) in accordance with label directions.

**Table 2:** Larval and adult control of *Aedes albopictus* within 200 m near imported Zika virus (ZIKV) patients’ residences during mosquito season in the Republic of Korea, 2016

Mosquito management*	Times†	Methods‡	Pesticides§
Surveillance	2 times/week	BG-2 sentinel	–
Larval control	1 time/week	Habitat removal, larvicide	SumiLarv 0.5G, Abate 200E, Bactosec
Adult control			
Thermal fogging	3 times/week	Potable thermal fogger	Vegadelta 25EC, Greenbug plus EC, Triplebifen
Residual spray	Once at 15 days	Potable residual sprayer	Etovega^®^ EC, Phantom smart^®^ EC

* Mosquito management was conducted for a period of 3 weeks near ZIKV patient residences.

† Number of vector monitoring and control for a period of time.

‡ BG-2 Sentinel mosquito trap (BioQuip products Inc., CA); Potable thermal fogger (SS-fog 150 or 180; Seshin industrial Co.); Potable residual sprayer (Clover air compressed sprayer TH-33).

§ Pesticides included one or more of the following methods: Larval control: SumiLarv 0.5G; Pyriproxifen 0.5%; Insect Growth Regulator, Abate 200E; Temephos 1%; Organophosphate, Bactosec; *Bacillus thuringiensis israelensis* 31.43%; Adult control: Vegadelta EC; deltamethrin 2.5%; Pyrethroid, Greenbug plus EC; etofenprox 5%; pyrethroid, Triplebifen: bifenthrin 5%; pyrethroid, Eto vega^®^ EC; etofenprox 10%; pyrethroid and Phantom smart^®^ EC; etofenprox 10%; pyrethroid.

Larval control was conducted weekly for 3 weeks by removing small artificial containers and water from plant pot bases or using larvicides, for example, Sumilarv^®^ 0.5G (0.5% pyriproxifen) (Catchers Co. Ltd., Kimhae, ROK), Bactosec^®^ [3,500 ITU/mg *Bacillus thuringiensis israelensis* (*Bti*)] (Sungin Pharma Co., Damyang, ROK), or Abate^®^ 200E (20% temephos; Pharmcle, Ansan, ROK) to discarded tires and nonremovable larval habitats within 200 m of patients’ residences in accordance with label directions. Thermal fogging was not conducted at the residence of ZIKV patient 8-1 because of a privacy request by the patient. Vector control at the residence of ZIKV patient 15 (confirmed ZIKV-positive in mid-October) was not conducted because surveillance did not result in the collection of *Ae. albopictus*.

### Data analysis.

Results from surveillance and control were entered into a database and analyzed using the SAS program. Mean values of population densities based on TIs before and after control for the six BG Sentinel traps located within 200 m of each of the residences of the ZIKV patients and with or without nearby forested areas were compared using the *T*-test in the SAS program.^14^ The mean values were determined by applying the following formula in the SAS program:

$$\mathrm{Mean collection value}=100-\frac{\text{Number of }\mathrm{Ae}\text{. }\mathrm{albopictus}\text{ in one trap}}{\text{Number of }\mathrm{Ae}\text{. }\mathrm{albopictus}\text{ in traps installed at six points}}$$

Control efficacy of *Ae. albopictus* was calculated for evaluation using the following formula:

Control efficacy=100−(Number of Ae. albopictus after controlNumber of Ae. albopictus before control)×100

## RESULTS

### Imported ZIKV cases in the ROK, 2016.

**Figure 1.**
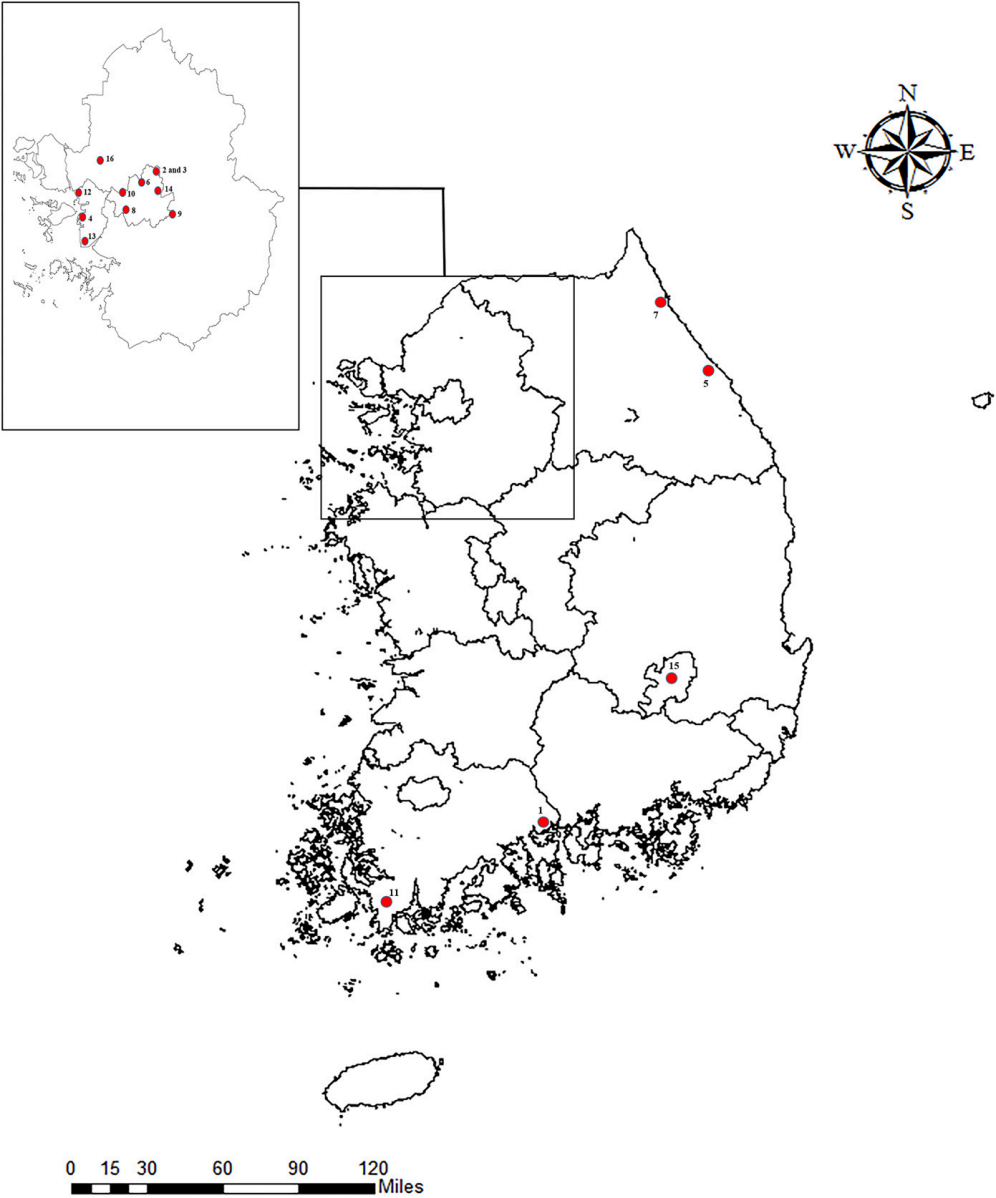
Distribution of imported Zika virus (ZIKV) cases in the Republic of Korea during 2016 as a result of travel to South/Central America, Caribbean, Southeast Asia, and the Pacific Islands. Numbers indicate ZIKV case numbers (see Table 4). This figure appears in color at www.ajtmh.org.

A total of 16 imported ZIKV cases (Philippines [6], Vietnam [4], Thailand [2], Brazil [1], Guatemala [1], Puerto Rico [1], and the Dominican Republic [1]) were identified in ROK residents that had traveled to ZIKV endemic areas in 2016. A total of nine cases were identified from June to September when *Ae. albopictus* populations are highest (Figure 1, Table 3). Based on patient interviews, infections in all cases were due to mosquito bites. All of the patients, except one asymptomatic case, demonstrated a rash, whereas 14 patients demonstrated one or more of the following symptoms: fever, myalgia, arthralgia, and conjunctivitis (Table 3). For eight of the ZIKV patients, ZIKV was detected in both blood and urine, but was only detected in urine (7) or blood (1) in the other eight cases.

**Table 3:** Imported Zika virus (ZIKV) cases from Southeast Asia, Africa, South and Central America, and the Caribbean into the Republic of Korea during 2016

Case number	Confirmation date*	Symptoms†,‡	Country of virus origin	Entry date	Forest area§	Detection	MM‖
6	June 30	Rs, Fv, Ar, My, Cj	Dominican Republic	June 23	Yes	Blood (−), urine (+)	L, TF, RS
7	July 9	Rs, Cj	Guatemala	July 6	No	Blood (+), urine (+)	L, TF, RS
8 (R) and 8 (O)¶	July 13	Rs, Ar	Puerto Rico	July 4	Yes	Blood (−), urine (+)	L, TF, RS and L, RS
9	July 28	Rs, Fv, Ar, My	Vietnam	July 15	No	Blood (+), urine (+)	L, TF, RS
10	August 19	Rs, Fv, My	Thailand	August 8	Yes	Blood (+), urine (−)	L, TF, RS
11	August 26	Rs, My	Vietnam	August 2	No	Blood (+), urine (+)	L, TF, RS
12	September 14	Rs, Fv, My, Cj	Philippines	September 6	No	Blood (+), urine (+)	L, TF, RS
13	September 17	Rs	Philippines	September 13	No	Blood (+), urine (+)	L, TF, RS
14	September 23	Rs, Ar, My	Thailand	September 16	No	Blood (−), urine (+)	L, TF, RS

* Date = date of laboratory confirmation.

† Symptoms: Ar = arthralgia; Cj = conjunctivitis; Fv = fever; My = myalgia; Rs = rash.

‡ Mosquito bites were the source of all infections based on an epidemiological investigation of each of the patients.

§ Forested areas within 200 m of a patient’s residence.

‖ L = larval control; MM = mosquito management included mosquito surveillance and control of *Aedes albopictus* for 3 weeks after detection of ZIKV; RS = residual spraying; TF = thermal fogging.

¶ Cases 8 (R) and 8 (O) are the same patient. Case 8 (R) represents case 8 residence and case 8 (O) represents case 8 office. Vector control was conducted only near the patient’s residence. Patient 8 (O) reported mosquito bites at his workplace, but only mosquito surveillance was conducted.

### Mosquito surveillance near residences of ZIKV patients before and after vector control.

Overall, a total of 9,410 mosquitoes, including 809 *Aedes* and 496 *Ochlerotatus* spp. were collected (Table 4). *Aedes albopictus* was collected in 8/10 residences surveyed where patients were diagnosed with ZIKV from June to September before the implementation of control measures (Table 4). For all residences located in forested and nonforested areas, *Culex pipiens* complex was the most frequently collected mosquito in BG Sentinel traps, accounting for 19.2–64.1% and 77.6–99.6% of all mosquitoes collected, respectively, followed by *Ae. albopictus* (19.2–41.0% and 0–7.8%), *Ochlerotatus koreicus* (0.7–20.3% and 0.03–6.2%), and *Culex tritaeniorhynchus* (0% and 0.03–85.8%). *Aedes albopictus* TIs within 200 m of residences near forested areas were significantly higher than those of residences where forested areas were absent. The mean ± SE values of mosquitoes collected in traps for residences located in/near forested and nonforested areas were 29.3 ± 6.50 and 3.1 ± 0.55 (*t*-value = 1.84, df = 12, *P* value = 0.0354). The overall mean number of *Ae. albopictus* collected near patient residences before and after vector control were 10.6 and 1.3, respectively, with an effective control rate of 83.1% (Table 5). The mean numbers of *Ae. albopictus* captured near patients’ residences near forested areas before and after vector control were 29.3 and 2.5, respectively, and without nearby forested areas were 3.1 and 0.8, respectively, with estimated control rates of 90.4% and 75.8% for residences in/near forested areas and without nearby forests, respectively.

**Table 4:** Trap indices for *Aedes albopictus* collected using six BG-2 Sentinel traps within 200 m of each of the Zika virus patients’ residences after the confirmation of Zika disease during the 2016 mosquito season and before vector control was implemented

Case number	Number collected mosquito species										Total
	*Ae. albopictus*	*Aedes vexans nipponii*	*Anopheles* spp.	*Armigeres subalbatus*	*Culex bitaeniorhynchus*	*Culex orientalis*	*Culex pipiens*	*Culex tritaeniorhynchus*	*Ochlerotatus koreicus*	*Ochlerotatus togoi*	
6 (forest)	137	17	0	0	0	0	415	0	78	0	647
7 (no forest)	16	55	115	11	2	24	3,350	1	1	6	3,581
8 (R) (no forest)	10	0	0	0	0	0	801	0	101	0	912
8 (O) (forest	240	0	0	0	0	0	342	0	4	0	586
9 (no forest)	30	8	0	21	0	0	307	0	19	0	385
10 (no forest)	215	17	0	0	0	0	661	0	227	0	1,120
11 (no forest)	0	21	0	0	0	0	0	223	16	0	260
12 (no forest)	21	5	3	69	0	8	499	7	31	0	643
13 (no forest)	17	0	1	0	0	0	715	2	11		746
14 (no forest)	0	0	0	0	0	0	528	0	2	0	530
Total	686	123	119	101	2	32	7,618	233	490	6	9,410

**Table 5:** Total number (mean and SE) of *Aedes albopictus* collected before and at 3 weeks after vector control using six BG-2 Sentinel traps operated for 24 hours, respectively, within 200 m of patient residences located near groves of trees/forested areas or without trees after confirmation of Zika virus (ZIKV) infections among returning travelers during the mosquito season (June to October), 2016

Case number*	Number *Ae. albopictus* (Mean ± SE)†		Control rates (%)‡ (*P* value)§
	Pre-treatment‖	Post-treatment¶	
Control			
8 (O)#	240 (40.0 ± 9.07a)	208 (34.7 ± 6.16a)	13.3 (0.4566)
Forest			
10	215 (35.8 ± 4.44a)	12 (2.0 ± 1.03b)	94.0 (0.0230)
6	137 (22.8 ± 1.87a)	18 (3.0 ± 1.04b)	87.0 (0.0002)
Mean (%, Mean ± SE)**	352 (29.3 ± 6.50a)	30 (2.5 ± 0.50b)	91.0 (0.0305)
No forest			
7	16 (2.7 ± 0.33a)	6 (1.0 ± 0.63a)	62.0 (0.0212)
8 (R)	10 (1.7 ± 0.75a)	2 (0.3 ± 0.15a)	80.0 (0.1729)
9	30 (5.0 ± 1.69a)	14 (2.4 ± 0.33b)	53.0 (0.1537)
12	21 (3.5 ± 0.92a)	1 (0.2 ± 0.17a)	95.0 (0.0039)
13	17 (2.8 ± 1.91a)	2 (0.3 ± 0.21a)	88.0 (0.2335)
Mean (%, Mean ± SE)	94 (3.1 ± 0.55a)	25 (0.8 ± 0.42b)	74.0 (0.0069)
Total (mean)**	446 (10.6 ± 5.04a)	55 (1.3 ± 0.43b)	82.5 (0.0354)

SE = standard error.

* *Aedes albopictus* were not collected at residences of ZIKV patients 11 and 14.

† Means within a row and column followed by the same letter are not significantly different (*P* < 0.05, *T*-test) (six replicates).

‡ Control rates = 100 − [(Number of *Ae. albopictus* after control/Number of *Ae. albopictus* before control) × 100].

§ *P* value significant at < 0.05.

‖ Before control: Number of *Ae. albopictus* collected in six traps during 1 day before control.

¶ After control: Number of *Ae. albopictus* collected in six traps during 1 day and 3 weeks after first collection.

# 8 (O): Forest area. No chemical control, such as residual spray and thermal fogging, was conducted due to personal information protection and type of office equipment.

** Total (mean): Overall total number collected and mean number collected for residences located in/near forests and residences located in areas without trees before and after treatement. Number of *Ae. albopictus* collected for case 8 (O) was not included.

Overall, mean TIs of *Ae. albopictus* for all residences were significantly lower after 3 weeks of treatment (5.5) when compared with pretreatment levels (14.3) (Table 5). However, while TIs were lower for all residences after treatment, there were no significant differences in the TIs for residences where the initial mean numbers of *Ae. albopictus* were < 5.0 or at the one residence where control measures were not implemented (Table 5). For patient 8 (R), the mean number of *Ae. albopictus* collected was 40.0, whereas at 3 weeks after only larval control and residual spray were conducted, the TI was not significantly reduced (34.7).

### Detection of ZIKV, DENV, and CHIKV.

A total of 686 *Ae. albopictus*, 123 *Ae. vexans nipponii*, and 490 *Oc. koreicus*, and six *Oc. togoi* were assayed for the presence of ZIKV, DENV, and CHIKV. None of the individually assayed specimens were positive for any of the viruses tested.

## DISCUSSION

On February 1, 2016, an Emergency Committee was convened by the Director General under the International Health Regulations (2005).^15^ Following the advice of the Committee, the Director General announced a recent cluster of microcephaly and other neurologic disorders reported in Brazil to be a Public Health Emergency of International Concern. The Emergency Committee agreed that a causal relationship between ZIKV infection during pregnancy and microcephaly was strongly suspected. The Director General emphasized that the most important protective measures were the implementation of mosquito control to reduce biting populations and the prevention of mosquito bites, for example, proper wearing of clothing and using effective repellents, especially for high-risk individuals, for example, pregnant women.

To prevent mosquito ZIKV transmission within 200 m of the confirmed ZIKV patients’ residences who had recently returned from ZIKV endemic areas, the KCDC, in collaboration with the LPHCs and LEHIs, conducted mosquito-borne disease surveillance and control during the mosquito season in accordance with the newly established guidelines, which demonstrated a relatively high level of efficacy for decreasing vector numbers.

Singapore focused on vector mosquito control from 1960 to 1970 to effectively prevent the spread of DENV that belonged to the family of viruses (Flaviviridae) as ZIKV, which are transmitted principally by *Aedes* species mosquitoes.^16^ Dengue fever (DF) appeared in Singapore and quickly became a major cause of childhood death as a result of a hemorrhagic syndrome. A public health response to DF began in 1966, when the Vector Control Unit was set up within the Quarantine and Epidemiology Branch, initially in the Ministry of Health but transferred to the Ministry of the Environment in 1972, when DF was made a notifiable disease in 1977.^17^ From 1966 to 1968, after a series of entomologic surveys^18–22^ and a pilot project to control *Aedes* vectors in an area with high incidence of DF,^23^ a vector control system based on entomologic surveillance and larval source reduction (i.e., reducing the availability of *Aedes* larval habitats) was developed and implemented in 1968.^17^ Program emphasis was placed on the elimination of mosquito breeding sites to reduce adult populations that precede disease transmission. The hypothesis was that controlling the vector populations before the disease was detected would result in the reduction of transmission. After the implementation of larval and adult *Ae. aegypti* control, Singapore experienced a 15-year period of low dengue incidence with < 10 cases annually.

In Japan, after a hiatus of 70 years without confirmed autochthonous cases of DF, 19 cases were reported.^24,25^ The response focused mostly on personal protection, whereas concurrently vector control and surveillance were implemented which showed that vector populations decreased as a result of control strategies.^25,26^

The TIs *for Ae. albopictus*, using the BG-2 Sentinel mosquito traps, were < 10 until May, when TIs rapidly increased more than 100-fold in June. *Aedes albopictus* numbers thereafter gradually increased through early September, but then decreased to low levels by late September/early October before disappearing with the onset of the winter season. *Aedes albopictus* larval control (removal of larval breeding habitats and use of larvicides) was conducted until May, whereas from June until the end of the mosquito season, both larval and adult control were implemented within 200 m of ZIKV patient residences for 3 weeks after the confirmation of ZIKV infections in ROK travelers. ZIKV is commonly detected in human blood for 3 days after the onset of symptoms, but can be detected in some cases for up to 14 days. While it is possible that *Ae. albopictus* may bite patients during the incubation period, we were not able to survey patients at the previremic stage in South Korea. After feeding on ZIKV infected blood, ZIKV disseminated from the midgut of all *Ae. albopictus,* and 73% of the mosquitoes had ZIKV in their saliva.^27^

In the present study, vector control was limited to within 200 m of patient residences, taking into consideration that the flight range of *Ae. albopictus* is typically < 200 m from the forests edge.^28–30^ The identification of the major habitats of *Ae. albopictus* in the ROK is a critical factor for efficient vector control. The mean TIs of *Ae. albopictus* within 200 m of ZIKV patient residences near and distant from forested areas were significantly lower after control measures were implemented. Although control residences (without vector control) at the one residence where vector control was not implemented, TIs were similar during the 3-week period after diagnosis of ZIKV infection, indicating that the implemented control measures had a significant effect on reducing vector populations.

During 2017, more intensive ZIKV, DENV, and CHIKV control programs, including vector populations for patient residences within 200 m of forests are planned. Thermal fogging of nearby bushes/forests demonstrated a high level of effective control for *Ae. albopictus* adults, most likely, because adults typically rest on the back of leaves in bushes and only emerge from the vegetation when a blood source approaches. In the present study, it was not possible to conduct thermal fogging for the control of adult *Ae. albopictus* near the office of patient 8 (O) out of respect for the patient’s privacy. However, other types of control, such as larval control and residual spraying, were conducted near the residences of other patients. Nevertheless, the control rate of *Ae. albopictus* near this patient’s residence was only 13.3%, which was significantly lower when compared with control rates near other patient residences. Although the population density of *Ae. albopictus* near the residences of patients 7, 8, 12, and 13 decreased after control, the control rates were not significantly different. This might be attributed to the low pretreatment *Ae. albopictus* TIs (< 5).

Although the overall mean control rate for *Ae. albopictus* was 87.7%, potential pesticide resistance should be assessed, thereby facilitating the selection of pesticides for more effective control. All *Aedes* and *Ochlerotatus* spp. collected were negative for ZIKV, DENV, and CHIKV. To date, there have been no reports of autochthonous transmission of ZIKV, DENV, and CHIKV in the ROK and only one event of autochthonous transmission of DENV in Japan.^10^ Accordingly, vector control is essential to efficiently prevent transmission of these three viruses during the summer mosquito season. While the KCDC guidelines for *Ae. albopictus* control (drafted in 2016) has proven effective for reducing vector populations and potential transmission of ZIKV near residences of ZIKV patients, stricter patient management, for example, increased hospitalization periods, to reduce the potential for transmission at their residences should be considered.

ZIKV vector management near Zika patients’ residences, conducted in accordance with guidelines for *Ae. albopictus* control established in 2016, significantly reduced vector populations when precontrol TIs were > 5. Thus, control measures implemented in accordance with the KCDC vector control guidelines may effectively reduce the potential for autochthonous transmission of not only ZIKV but also DENV, and CHIKV in the ROK. Although ZIKV, DENV, and CHIKV have not been detected in *Aedes* spp. or other potential vectors in the ROK, the KCDC must remain vigilant by monitoring patients with suspected mosquito-borne diseases to reduce the potential of viral transmission to resident populations. Arbovirus vector control can be effectively accomplished through the strengthening of national and local government relationships and coordination for the implementation of effective vector control strategies.
